# Development and Evaluation of Topical Zinc Oxide Nanogels Formulation Using *Dendrobium anosmum* and Its Effect on Acne Vulgaris

**DOI:** 10.3390/molecules28196749

**Published:** 2023-09-22

**Authors:** Yu Yang Tan, Ling Shing Wong, Kar Lin Nyam, Kitiyaporn Wittayanarakul, Nurliyana Ahmad Zawawi, Kavitha Rajendran, Sinovassane Djearamane, Anto Cordelia Tanislaus Antony Dhanapal

**Affiliations:** 1 Department of Chemical Science, Faculty of Science, Universiti Tunku Abdul Rahman, Kampar 31900, Malaysia; tanyuyang111@gmail.com; 2 Life Science Division, Faculty of Health and Life Sciences, INTI International University, Nilai 71899, Malaysia; 3 Department of Food Science and Nutrition, Faculty of Applied Sciences, UCSI University, Kuala Lumpur 56000, Malaysia; nyamkl@ucsiuniversity.edu.my; 4 Program in Science Technology and Business Enterprise, Faculty of Interdisciplinary Studies, Khon Kaen University, Nong Khai Campus, Nong Khai 43000, Thailand; kitiwi@kku.ac.th; 5 Department of Bioscience, Faculty of Science, Universiti Teknologi Malaysia, Johor Bahru 81310, Malaysia; nurliyana@utm.my; 6 Department of Pharmaceutics, SRM Institute of Science and Technology, SRM College of Pharmacy, Kattankulathur 603203, Tamil Nadu, India; kavithar@srmist.edu.in; 7 Department of Biomedical Science, Faculty of Science, Universiti Tunku Abdul Rahman, Kampar 31900, Malaysia; sinouvassane@utar.edu.my

**Keywords:** biogenic synthesis, *Cutibacterium acne*, FE-SEM, FTIR, hybrid nanogel, phytochemicals, UV-Vis, XRD, zinc oxide nanoparticles, green product

## Abstract

Zinc oxide nanoparticles have high levels of biocompatibility, a low impact on environmental contamination, and suitable to be used as an ingredient for environmentally friendly skincare products. In this study, biogenically synthesized zinc oxide nanoparticles using *Dendrobium anosum* are used as a reducing and capping agent for topical anti-acne nanogels, and the antimicrobial effect of the nanogel is assessed on *Cutibacterium acne* and *Staphylococcus aureus*. *Dendrobium anosmum* leaf extract was examined for the presence of secondary metabolites and its total amount of phenolic and flavonoid content was determined. Both the biogenically and chemogenic-synthesized zinc oxide nanoparticles were compared using UV-Visible spectrophotometer, FE-SEM, XRD, and FTIR. To produce the topical nanogel, the biogenic and chemogenic zinc oxide nanoparticles were mixed with a carbomer and hydroxypropyl-methyl cellulose (HPMC) polymer. The mixtures were then tested for physical and chemical characteristics. To assess their anti-acne effectiveness, the mixtures were tested against *C. acne* and *S. aureus*. The biogenic zinc oxide nanoparticles have particle sizes of 20 nm and a high-phase purity. In comparison to chemogenic nanoparticles, the hydrogels with biogenically synthesized nanoparticles was more effective against Gram-positive bacteria. Through this study, the hybrid nanogels was proven to be effective against the microbes that cause acne and to be potentially used as a green product against skin infections.

## 1. Introduction

Green nanotechnology is a rapidly evolving field that aims to address the development towards environmentally sustainable nanotechnology applications. The divergence in plants, peculiarly in traditional herbal plants, has attracted research for their utilization for centuries [1]. *Dendrobium* spp. has been long used in traditional Chinese medicine (TCM) and has a significant variation in secondary metabolites, which possess a strong defense mechanism from pathogenic attacks and also benefits humans with the bioactive constituents they possess [2]. In addition to its ornamental value, the *D. anosmum* plant holds significant medicinal importance among the local communities across Southeast Asia. The uniqueness of the various metabolites found in *D. anosmum* aid the enhancement in metallic nanoparticle (NP) maturation, which has piqued further interest in the employment of green-synthesized products in various applications [3]. The development of biogenic metallic nanoparticles was accomplished by the utilization of plant extracts with a comparatively higher efficiency source and technical mass production from fungi, algae, or bacteria [4]. Green-synthesized products have been proven to lower potentially toxic impacts by eliminating the traditional use of chemicals and solvents [5].

Acne vulgaris is a chronic inflammatory and self-limiting skin disorder resulting from the abnormal keratinization of the pilosebaceous unit (hair follicles alongside with sebaceous gland), bacterial colonization, high sebum production, genotypic influence, and hormonal changes [6]. In Malaysia, due to the high consumption of dairy and glycemic-load diets, statistical analysis indicated that 1 in 2 citizens, from adolescents to young adults, shows signs of acne vulgaris skin disorder [7,8]. At present, treatments focus on antibiotics to proclaim antibacterial effects by limiting the main acne-triggering bacteria, belonging to the *Cutibacterium acne*, *Malassezia furfur*, and *Staphylococcaceae* family [9,10]. However, due to the misuse and overuse of antibiotics, antimicrobial resistance (AMR) has been classified as the most consequential health threat in the 21st century [11]. Interest towards ZnO NPs has been growing since the discovery of their antibacterial properties in recent years and are intended as alternatives to antibiotics. They are well known for their low toxicity, UV-absorption rate, and biodegradability, making them an excellent candidate in the biomedical field [12]. The penetration pathway of ZnO NPs into the stratum corneum or the accumulation of zinc ions within skin folds or roots of the hair follicle shows a lack of penetration of ZnO NPs into the viable epidermis that might lead to a toxic response [13,14]. The incorporation of metallic nanoparticles in nanogel polymers has been proven to be more biocompatible as anti-acne treatment medicine than the present anti-acne gelling agent [15,16]. It has been suggested that the incorporation of nano-sized materials into polymer matrices will result in a hybrid composite material and exhibit enhanced antibacterial activity properties [17].

In this work, we screen *Dendrobium anosmum* for secondary metabolites to highlight the beneficial properties of ZnO NPs using chemogenic and biogenic methods. The synthesized ZnO NPs were incorporated in different types of polymers (carbomer and HPMC), forming a topical antibacterial nanogel. The study aims to establish the antibacterial efficacy of ZnO NPs and topical nanogels against Gram-positive (*Cutibacterium acne* and *Staphylococcus aureus*) bacteria. The results of this study can be considered as a preliminary step towards developing a novel pharmaceutical anti-acne agent utilizing eco-friendly nanomaterials. 

## 2. Materials and Methods

### 2.1. Preparation of the Plant Extract

The extraction process was conducted referring to [18]. *Dendrobium anosmum* Lindl. leaves were freshly picked from the mother plant and rinsed under running tap water; then, they were air-dried under indirect sunlight conditions. The dried leaves were ground into a powder using an electrical grinder. By using an aqueous extraction technique, 4 g of the leaf powder was mixed with 100 mL of deionized water and heated to 70 °C for 20 min. The plant extract was filtered using Whatman filter paper no. 1 (filtraTech, Saint-Jean-de-Braye, France) and was stored for further analysis.

### 2.2. Phytochemical Screening

To determine the phytochemicals or secondary metabolites in the plant extract of *Dendrobium anosmum*, preliminary screenings for alkaloids [19], carotenoid [20], coumarin [21], flavonoids [22,23], phenols [24], phlobatannin [25], saponin [26], steroid [27], tannins [28], and terpenoids [29] were performed.

#### 2.2.1. Determination of the Total Phenolic Content

The total phenolic content for plant extract was measured using the Folin–Ciocalteu assay (Sisco Research Laboratories Pvt. Ltd, Mumbai, India) as described by Dhanapal and Azlim Almey [30,31]. Gallic acid was used as the standard by preparing 1 mg/mL using methanol as the solvent. The stock solution was diluted with deionized water, forming a working concentration ranging from 0.02 mg/mL to 0.14 mg/mL. The plant extract with a volume of 100 μL was added with 750 μL of 10% Folin–Ciocalteu reagent and then incubated in the dark at room temperature. A 750 μL of 6% sodium carbonate (Na_2_CO_3_) was added into the incubated solution and mixed gently. The solution was kept in the dark for 90 min before taking the absorbance reading at 760 nm using the UV-Vis spectrophotometer (Thermo Scientific GENESYS 10S, Thermo Fisher Scientific, Waltham, MA, USA). The total phenolic content was expressed in mg of gallic acid equivalents (GAE)/g of dry matter with reference to the gallic acid standard calibration curve. 

#### 2.2.2. Determination of the Total Flavonoid Content

The total flavonoid content was determined using the aluminum chloride method described by Samanta [32]. Quercetin hydrate with 1 mg/mL was prepared as a standard stock solution. Methanol was used as a solvent for stock dilution into a working concentration between 0.2 mg/ mL and 1 mg/mL. Then, 100 μL of plant extract was added to sodium nitrite (NaNO_2_) at a 5% concentration. The mixture was then incubated at room temperature for approximately 6 min. A volume of 150 μL with 10% aluminum chloride hexahydrate (AlCl_3_·6H_2_O) was prepared and added to the mixture, and then incubated at room temperature for 6 min. Following that, 800 μL of 10% NaOH was added and mixed thoroughly before absorbance was taken at 510 nm using UV-Vis spectrophotometer. The total flavonoid content was expressed in mg of quercetin equivalents of dry matter, referring to the quercetin hydrate standard calibration curve. 

### 2.3. Synthesis of the Zinc Oxide Nanoparticles

#### 2.3.1. Chemogenic Synthesis of Zinc Oxide Nanoparticles

The chemogenic synthesis of zinc oxide nanoparticles was conducted using the co-precipitating method by Chikere [33]. Chemically synthesized zinc oxide nanoparticles (C-ZnO NPs) was synthesized by completely dissolving 12 g of zinc nitrate hexahydrate (Zn(NO_3_)_2_·6H_2_O) in 100 mL of deionized water in a beaker with a magnetic stirrer for 25 min. The constant stirring continued as the heating process reached a temperature of 70 °C. Sodium hydroxide (NaOH) with 3.2 g weight was prepared and dissolved in 30 mL deionized water in a separate beaker with approximately 10 min of stirring. The drop-wise technique was applied for NaOH addition into (Zn(NO_3_)_2_·6H_2_O) under continuous stirring. The mixture was maintained at 70 °C for 2 h with constant stirring until a white suspension was formed that confirmed the synthesis process. The solution was left to cool and was filtered using Whatman filter paper no. 1. The filtered sample was transferred to a ceramic crucible for furnace calcination at 450 °C for 2 h. 

#### 2.3.2. Biogenic Synthesis of Zinc Oxide Nanoparticles

The green zinc oxide nanoparticle synthesis technique was proposed by Basnet [34]. It was initiated by heating 50 mL of the plant extract at 60 °C. Zinc nitrate hexahydrate (Zn(NO_3_)_2_·6H_2_O), at a weight of 2 g, was added into the hot plant extract with continuous stirring. The reaction mixture was maintained at a temperature between 60 °C and 70 °C until the formation of approximately 5 mL of a blackish-brown solution, indicating that the synthesis of green ZnO NPs (G-ZnO NPs) was accomplished. The resultant solution was calcinated at 450 °C for 2 h.

### 2.4. Characterization of the ZnO NPs

The morphology and structure of ZnO NPs were analyzed using varied analytical instruments [35,36]. The preliminary characterization of the absorption spectrum was recorded by the UV-visible Spectrophotometer (Thermo Scientific GENESYS 10S, Thermo Fisher Scientific, Waltham, MA, USA). FESEM (JEOL JSM-6701F, JEOL, Tokyo, Japan) was used to obtain the morphological structure and element composition. The X-ray Diffraction analysis was conducted using Shimadzu XRD 6000 (Shimadzu, Kyoto, Japan) with Cu Kα (λ = 1.5406 Å) radiation in a 2*θ* range from 10° to 80°. The FTIR (Perkin Elmer RX1 spectrophotometer, Perkin Elmer, Waltham, MA, USA) analysis was conducted in a range of 400–4000 cm^−1^ with a resolution of 4 cm^−1^ by using KBr pellets (Fisher Scientific, Waltham, MA, USA).

### 2.5. Formulation of the Nano-sized Topical Gel

The cold mechanical method described by Prabu [37] was employed for the nano-sized topical gel formulation. The 2 polymers used were Carbopol-940 and Hydroxypropyl Methylcellulose (HPMC). A total of 2 g of each polymer was sprinkled uniformly on the surface of 80 mL of deionized water. The mixture was left overnight for the complete absorption of the polymers. The concentration of chemogenic and biogenic ZnO NPs was set to 16 mg/mL. A total of 2 g of glycerol was added to the nanogels, followed by the addition of deionized water to a total of 100 mL.

### 2.6. Physiochemical Evaluation

The physical state of the topical gel was determined by several parameters, namely color, appearance, and consistency [37]. Moreover, a pH adjustment to ∼pH 5.5 was conducted to suit a healthy individual skin pH. Furthermore, the viscosity test was conducted using Viscometer (Brookfield DV2T, AMETEK Brookfield, Middleboro, MA, USA) with spindle no. 7. The spreadability test was determined by separating 2 standard glass slides with 6 cm in dimension and 40 g in weight, with the absence of an external force attached to a glass slide with gel sandwiched in between. The time required for the upper slide to move from the bottom was measured. 

### 2.7. Bacterial Strains and Growth Conditions

*Cutibacterium acne* (ATCC 11827) and *Staphylococcus aureus* (ATCC 29213) were used in this study. The *C. acne* strain was grown in anaerobic conditions on Columbia Agar w/5% sheep blood (CBA; Isolac, Shah Alam, Malaysia), and the *S. aureus* strain was grown on Mueller–Hinton (MH) agar [38,39]. A single colony was transferred and resuspended in 0.85% saline solution to a final cellular concentration of 0.5 McFarland turbidity standard suspension.

### 2.8. Anti-Acne Efficacy Assay

The anti-acne effectiveness of pure zinc oxide nanoparticles (C-ZnO NPs and G-ZnO NPs) and nanogels (Carbopol and HPMC) were evaluated using the agar well diffusion method [39,40]. A saline suspension of *C. acne* at 0.5 mL was spread evenly on CBA; then, wells with a diameter of 8 mm were punctured using a cork borer. ZnO NP solutions (0.1 mL) with different concentrations (2, 4, 8, and 16 mg/mL) were pipetted into the wells. Nanogels incorporated with 16 mg/mL ZnO NPs were pipetted at the volume of 0.1 mL into the wells. The agar plates were incubated at 37 °C anaerobically for 24 h. The diameter zone of inhibition was measured after the incubation period. A similar protocol was applied to *S. aureus* by using MH agar and incubated under aerobic conditions. 

### 2.9. Statistical Analysis

Microsoft Excel 365 for Microsoft 365 MSO (Version 2306)was used to perform the statistical analysis of the data. The datasets were obtained in triplicate from multiple samples, and the values are reported as mean ± standard deviation (SD). A one-way ANOVA was used to assess the significant differences between values of the zone of inhibition shown against both bacteria. A statistical significance level of *p* ≤ 0.05 was utilized to ascertain the occurrence of statistically significant results.

## 3. Results and Discussion

### 3.1. Phytochemical Screening

The phytochemical analysis revealed the presence of secondary metabolites in the plant extract, having a potential therapeutic and physiological effect. The phytochemical constituents detected in the *Dendrobium anosmum* aqueous extracts are carotenoids, coumarin, flavonoids, phenols, saponin, steroids, tannins, and terpenoids, with the absence of alkaloids and phlobatannin compounds, as shown in Table 1. The phytoconstituents in the plant extract studied, primarily phenols and flavonoid, have been documented to act as reducing and capping agents in the ZnO NP biosynthesis process. This reflects *D. anosmum*’s suitability as a biogenic ZnO NP synthesis candidate [41,42]. Furthermore, *D. anosmum*’s pharmacological significance was demonstrated with the presence of secondary metabolites that are responsible for its therapeutic capabilities [43].

### 3.2. Total Phenolic Content and Total Flavonoid Content

The determination of the total phenolic content (TPC) and total flavonoid content (TFC) of the *D. anosmum* leaf extract was determined using aqueous extraction. The total phenolic and flavonoid contents are often linked with the result of phytochemical studies. However, their concentrations remain unknown. The total phenolic content determined from the plant extract was 15.125 ± 0.18 mg GAE/g dry matter (standard curve equation: y = 3.1661x − 0.0564, r^2^ = 0.961). Additionally, the total flavonoid content determined was 13.101 ± 0.13 mg QE/g dry mass (standard curve equation: y = 0.64x + 0.2226, r^2^ = 0.976). This shows that there is a higher total phenolic content than total flavonoid content, as flavonoids are naturally occurring polyphenolic secondary metabolites in plants [44]. The results obtained show a lower extraction efficiency by comparing to other extraction techniques [45,46,47]. The presence of phenolic and flavonoid contents additionally indicates the possibility of the utilization of *D. anosmum* extract in zinc oxide nanoparticle synthesis, despite the polar nature of water limiting the solubility of non-polar, hydrophobic compounds and the lack of selective extraction [48].

### 3.3. Characterization of ZnO Nanoparticles

#### 3.3.1. Yield of ZnO Nanoparticles 

Table 2 compares the yield of nanoparticles produced by chemogenic and biogenic processes. It was found that the biogenic synthesis of ZnO was able to produce a higher yield than that of the chemogenic protocol with values of 69.75 ± 0.50% and 15.99 ± 0.25%, respectively. The green-synthesized ZnO NP possesses a higher yield, indicated by weight. Several factors affecting the low-weight yield of chemical synthesis are the formation of toxic by-products during the synthesis process, which reduces the yield of the desired product, and the multiple-step synthesis process, such as centrifugation and filtration, which increases the chances of weight loss during each step [49]. Biogenically synthesized nanoparticles is desired for an upscale synthesis because of its higher yield and biocompatibility than the chemically synthesized product [50].

#### 3.3.2. UV-Vis Spectroscopy Analysis

**Figure 1:**
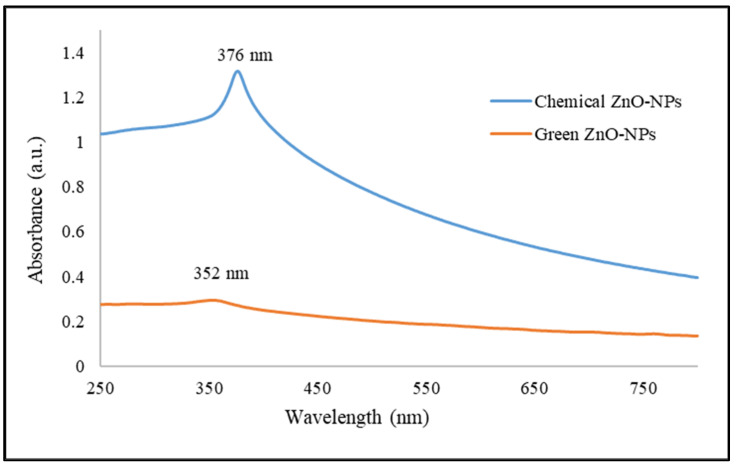
UV-Vis spectrum of the chemically and green-synthesized ZnO nanoparticles.

UV-Vis spectrophotometer provides the absorption spectra of both chemogenically synthesized zinc oxide nanoparticles (C-ZnO NPs) and biogenically synthesized ZnO NPs (G-ZnO NPs), as shown in Figure 1. An observable redshift absorption peak at 376 nm was obtained from the chemically synthesized ZnO NPs; the absorption peak of biogenically synthesized ZnO NPs was detected at 352 nm with a less intense peak. The presence of ZnO NPs in both samples was determined to be in the range of 350–380 nm using the same concentration of 1 mg/mL ZnO NPs dispersed in deionized water. Due to its lower absorption peak observed, the G-ZnO NPs can be regarded to form in a smaller size than that of chemogenically synthesized. UV-Vis detection for nanoparticles relies on observing peaks corresponding to electronic transitions spanning from the valence band to the conduction band [51]. The less intense absorption peak in biogenically synthesized ZnO NPs is caused by the aggregation of nanoparticles, which leads to an electronic structure change; therefore, a broader, less intense peak was observed. 

**Figure 2:**
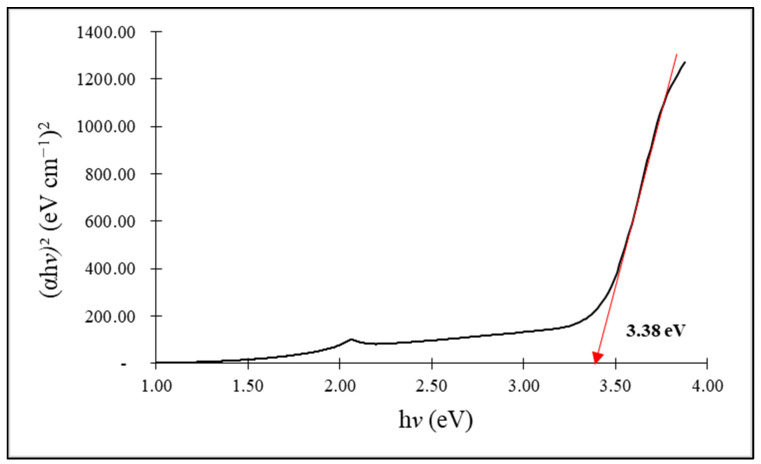
Band gap spectra of the chemically synthesized ZnO nanoparticles.

**Figure 3:**
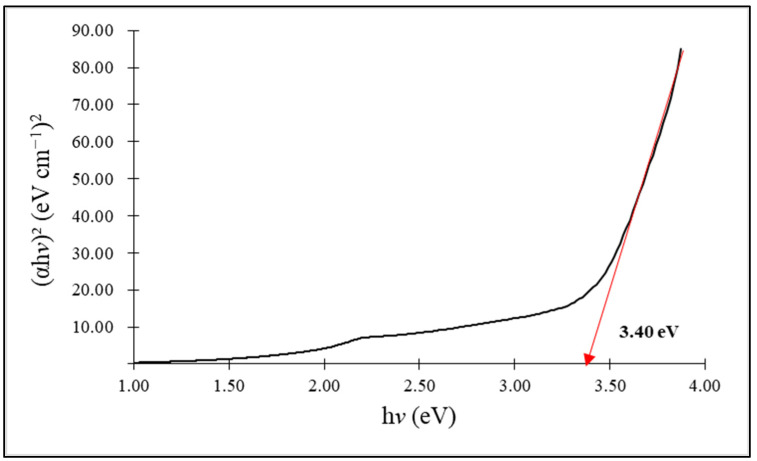
Band gap spectra of the green-synthesized ZnO nanoparticles.

Figure 2 and Figure 3 show the energy band gaps of the synthesized ZnO NPs, respectively, at 3.38 eV and 3.40 eV, for chemogenically synthesized ZnO nanoparticles (C-ZnO NPs) and biogenically synthesized ZnO nanoparticles (G-ZnO NPs). An average bulk ZnO is 3.37 eV lower than both synthesized ZnO NPs [52]. The relationship between the observed band gap energy and the nanoparticle size follows an inverse proportionality, consistent with the finding that G-ZnO NPs exhibit smaller crystalline sizes compared to C-ZnO NPs. 

#### 3.3.3. Morphological Analysis

**Figure 4:**
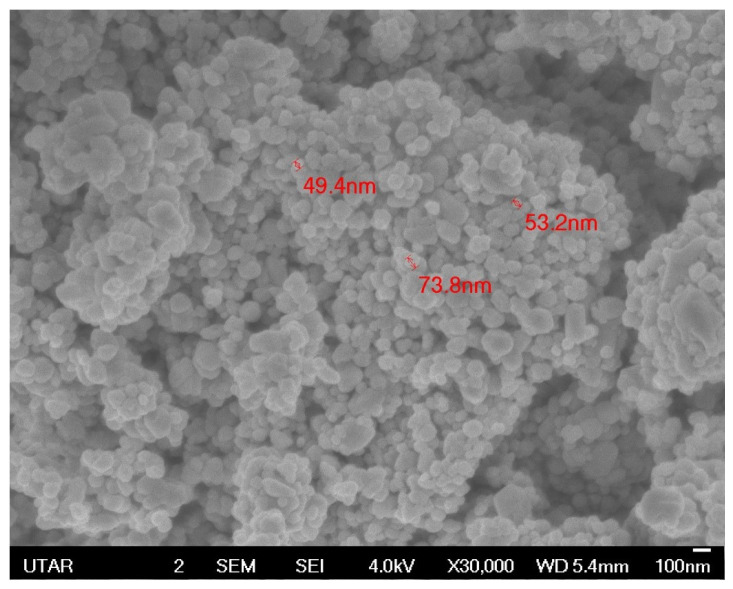
Morphology of the chemogenically synthesized ZnO NPs.

**Figure 5:**
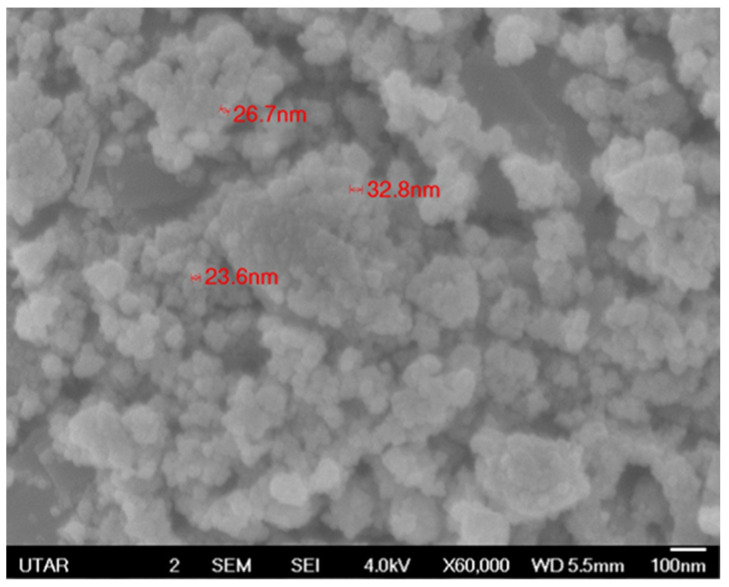
Morphology of the Dendrobium anosmum lead extract biogenically synthesized ZnO NPs.

The field emission scanning electron microscope (FE-SEM) shows the morphology and size of C-ZnO NPs and G-ZnO NPs. Figure 4 depicts the SEM image of C-ZnO NPs; the spherical shape of the ZnO nanoparticles was observed with an average size of 58.8 nm at ×30,000 magnification. Figure 5 shows the SEM image of G-ZnO NPs, showing a mixed composition with the majority being particles of spherical shape with an average size of 27.7 nm at ×60,000 magnification. The aggregation behavior can be observed more in the biogenically synthesized ZnO NPs than in the chemogenically ZnO NPs. Regarding the sizes of both nanoparticles, the biogenic ZnO nanoparticles had a smaller particle size than the chemically synthesized ZnO nanoparticles. The morphology difference can be ascribed to the utilization of natural reducing and capping agents, including phenolic compounds, saponin, alkaloids, flavonoids, terpenoids, and carbohydrates, instead of the chemically manufactured sodium hydroxide solution (NaOH) [53]. The salt precursor and calcination temperature remained the same in both synthesis processes; therefore, it is suggested that capping agents contributed to the variation of the particles’ shape and size.

#### 3.3.4. Crystalline and Structural Analysis

**Figure 6:**
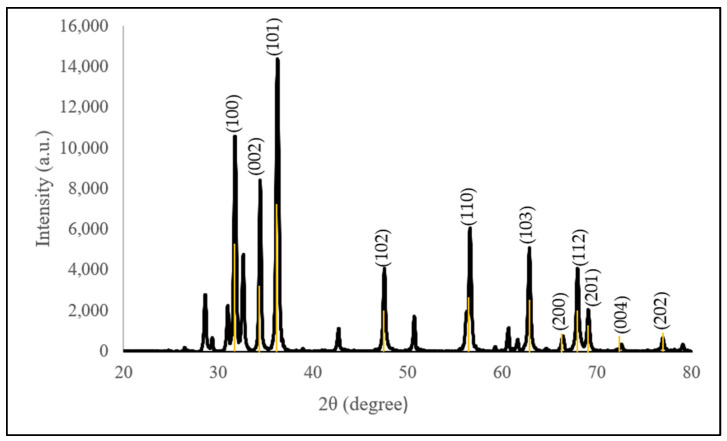
XRD pattern of the chemogenically synthesized ZnO NPs with the standard ICDD 01-070-8070.

**Figure 7:**
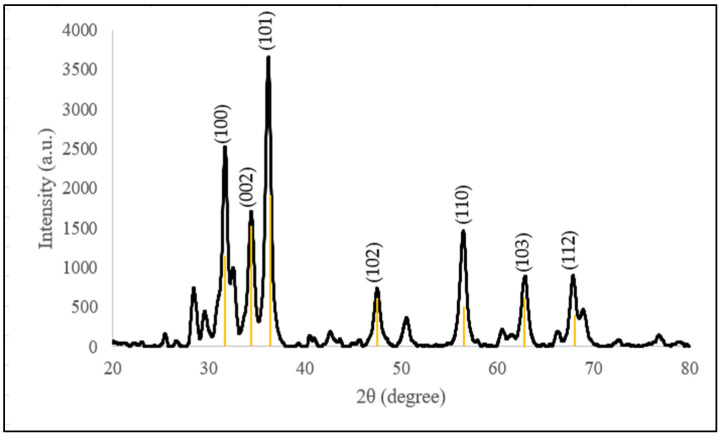
XRD pattern of the *Dendrobium anosmum* extract biogenically synthesized ZnO NPs with the standard ICDD 01-078-4493.

Table 3 shows the unit cell and crystalline size of C-ZnO NPs and G-ZnO NPs, while Figure 6 and Figure 7 show the XRD pattern, respectively, for ZnO NPs using different synthesis techniques. The pattern of the synthesized C-ZnO NPs was aligned with that in the International Centre for Diffraction Data, ICDD: 01-070-8070; that of the G-ZnO NPs was aligned with ICDD: 01-078-4493. The phase purity obtained in both samples was 95.6% and 95.5%, respectively. All synthesized ZnO NPs had hexagonal wurtzite structures, which are reported in other studies [54,55,56,57]. The average crystalline size was determined using Debye–Scherrer’s equation:

$$D=\frac{0.94\lambda }{\beta cos\theta }$$

Despite the diffraction differences, the peaks corresponding to the presence of impurities were absent, reflecting that pure nanoparticles were synthesized. Three additional peaks were observed in C-ZnO NPs, evidenced by the determination of higher-purity ZnO NPs.

#### 3.3.5. Fourier-Transform Infrared Functional Group Determination

**Figure 8:**
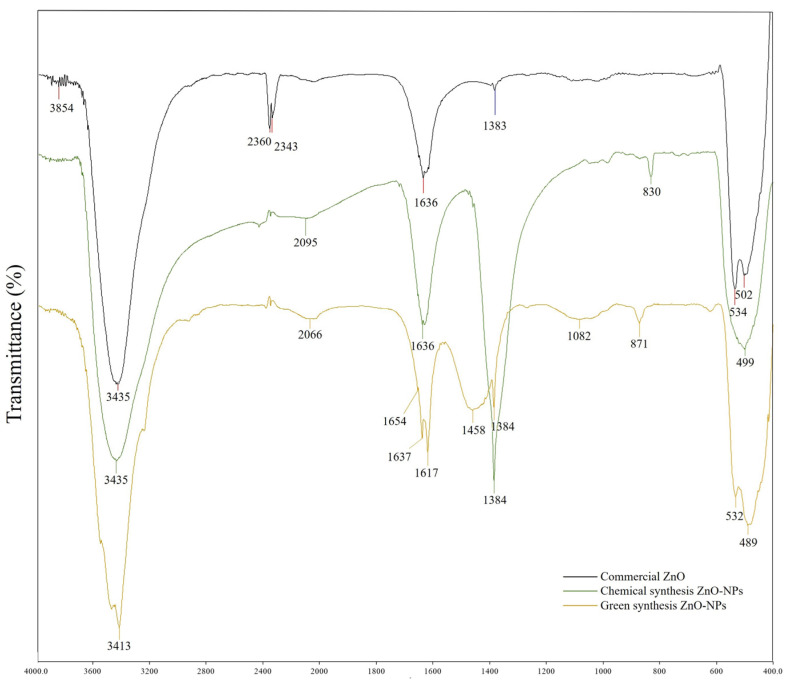
FTIR spectra of commercial and chemogenically and biogenically synthesized ZnO NPs.

The FTIR spectra recorded in Figure 8 and Table 4 show a comparison study between the functional groups within the three samples, which include commercial ZnO (Sime Scientific), C-ZnO NPs, and G-ZnO NPs. Bands around 3435 cm^−1^ were observed in all three samples, which refer to a hydroxyl group (O-H) stretch vibration. This can be attributed to the presence of water molecules on the ZnO surface as well as environmental influences [58]. Two additional bands were identified only in the commercial ZnO at the wavelengths of 2360 cm^−1^ and 2343 cm^−1^ and were assigned to the gas-phase CO_2_ molecules in the air [59,60]. The peak, however, was absent in the G-ZnO sample. Another observation includes the N=N=N asymmetric stretching that was observed around 2065 cm^−1^ in both C-ZnO NPs and G-ZnO NPs, which corresponds to a possible nitrogen source from the salt precursor of zinc nitrate hexahydrate [61]. Furthermore, the carbonyl group stretching (C=O) around 1636 cm^−1^ resulted from the band formation in all three nanoparticles. A similar band was determined at 1384 cm^−1^ in all three samples, which are attributed to the presence of the asymmetric stretching of nitrate ion (NO_3_^−1^) bending [62]. An absorption band at 1082 cm^−1^ was observed only in G-ZnO NPs, which is assigned to the alkane (C-C) stretching of the phytochemical residues on the surface of the nanoparticles [63]. In both C-ZnO NPs and G-ZnO NPs, a (C-H) stretching was observed at the wavelengths of 830 cm^−1^ and 871 cm^−1^, respectively [64]. The absorption bands around 530 cm^−1^ and 489 cm^−1^ correspond to the (Zn-O) stretching vibration of metal–oxygen. This band reflects the presence of ZnO NPs and the quantity of the nanoparticles for further characterization [65]. The FTIR screening reflects the functional groups’ biomolecules of the leaf extracts and the phytochemicals, which aids the reducing and capping during the ZnO NPs formation. These secondary metabolites help to prevent the agglomeration of NPs in an aqueous medium [66].

### 3.4. Physiochemical Evaluation of the Topical Nanogel

**Figure 9:**
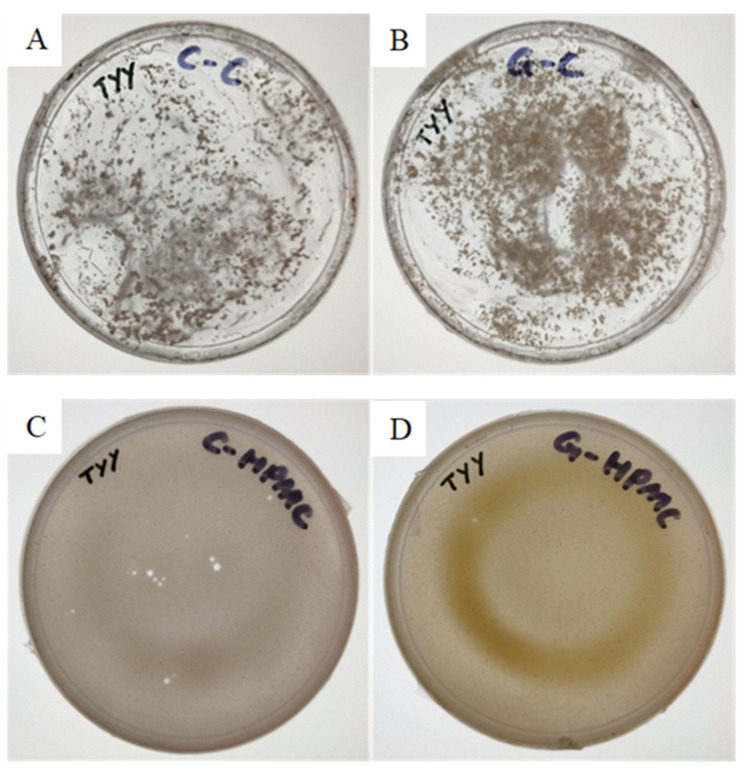
Image showing the physical appearance of the polymers incorporated with the synthesized ZnO NPs at a concentration of 16 mg/mL. (**A**) Carbopol gel with C-ZnO NPs. (**B**) Carbopol gel with G-ZnO NPs. (**C**) C-HPMC gel with C-ZnO NPs. (**D**) HPMC gel with G-ZnO NPs.

Table 5 shows the topical nanogel using Carbopol 940 and HPMC that was produced with several differences. Carbopol 940 prior to being incorporated in ZnO NPs is a transparent gel and HPMC is a semi-transparent gel; both gels are free from lumps, impurities, and composition-dependent, resulting in a different presentation. The appearance of both hybrid nano-sized gels differs with the incorporation of 16 mg/mL of C-ZnO NPs and G-ZnO NPs, respectively, forming C-Carbopol (chemogenically synthesized ZnO NPs incorporated in Carbopol 940), G-Carbopol (biogenically synthesized ZnO NPs incorporated in Carbopol 940), C-HPMC (chemogenically synthesized ZnO NPs incorporated in HPMC), and G-HPMC (biogenically synthesized ZnO NPs incorporated in HPMC). Significant differences in appearance were observed, despite both ZnO NPs being white powders. The Carbopol 940 gel remains as transparent gel with visible white nanoparticles observed within the gel, while HPMC shows a homogenized distribution of NPs over the gel, as shown in Figure 9. G-HPMC is a cream color gel that can be considered to have phytochemicals in the nanoparticles in the HPMC gel. The consistency of the Carbopol 940 gel is thick and smooth compared to that of the HPMC gel with a slender, smooth uniform texture. The viscosity of the Carbopol gel was determined to be much higher, at 45,386 ± 8.29 centipoises, than that of the HPMC gel, with 20,000 ± 2.16 centipoises. Regarding the determined viscosity, the HPMC gel shows a higher spreadability at 85.23 ± 0.19 g·cm/s and the Carbopol 940 gel has 6.56 ± 0.24 g·cm/s. The viscosity and spreadability are inversely proportional, and the observation of the HPMC gel is comparable with the result obtained in [37]. The Carbopol 940 gel exhibits a higher viscosity due to the pH adjustment. Both gels were adjusted to a pH of ~5.5, close to a healthy skin pH, with high agreement on the biophysical parameters of barrier function, scaling, moisturization, and ensuring the survival of skin microflora [67,68]. The viscosity of the Carbopol 940 gel is controlled by the neutralization of the aqueous dispersion in the range of around 40,000–60,000 centipoises, aligned with the result obtained by Ismail [16].

### 3.5. Anti-Acne Efficacy

**Figure 10:**
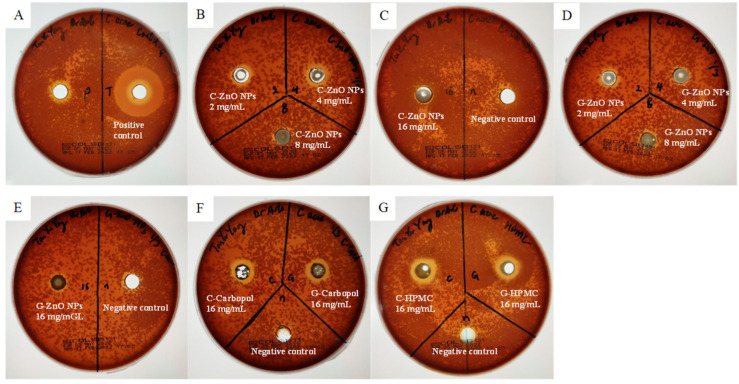
Image showing the antibacterial zone of inhibition (ZOI) of ZnO NPs and nanogels against *Cutibacterium acne*. (**A**) Positive control; (**B**) C-ZnO NPs at 2, 4, and 8 mg/mL; (**C**) C-ZnO NPs at 16 mg/mL and negative control; (**D**) G-ZnO NPs at 2, 4, and 8 mg/mL; (**E**) G-ZnO NPs at 16 mg/mL; (**F**) C- and G-Carbopol and negative control; and (**G**) C- and G-HPMC and negative control.

**Figure 11:**
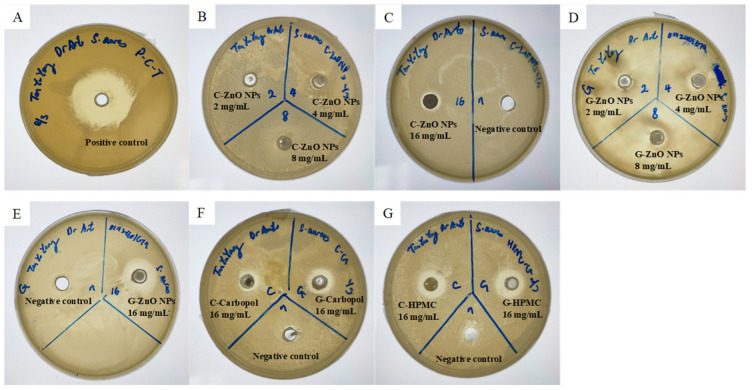
Image showing the antibacterial zone of inhibition (ZOI) of ZnO NPs and nanogels against *Staphylococcus aureus*. (**A**) Positive control; (**B**) C-ZnO NPs at 2, 4, and 8 mg/mL; (**C**) C-ZnO NPs at 16 mg/mL and negative control; (**D**) G-ZnO NPs at 2, 4, and 8 mg/mL; (**E**) G-ZnO NPs at 16 mg/mL; (**F**) C- and G-Carbopol and negative control; and (**G**) C- and G-HPMC and negative control.

The efficacy of the anti-acne activity by ZnO NPs and nanogels was evaluated using *C. acne* and *S. aureus* by using positive (tetracycline, 30 µg/mL) and negative (deionized water) controls [69]. Through our observation in Table 6, it can be seen that both the ZnO NP and nanogel samples show an inhibition zone in both *C. acne* and *S. aureus*, with 26 ± 1 mm and 33 ± 1 mm, respectively. The results of the antibacterial activity of all the samples are summarized in Figure 10 and Figure 11, and the efficacy was best observed in the order of G-Carbopol > C-Carbopol > G-HPMC > C-HPMC > G-ZnO NPs > C-ZnO NPs. The trend of the zone of inhibition observed against *C. acne* did not follow the proportional concentration rule. Both C-ZnO NPs and G-ZnO NPs showed an increase in the zone of inhibition, which was reduced as the concentration reached 16 mg/mL. The maximum zone of inhibition recorded for C-Zn NPs and G-ZnO NPs was 4 mg/mL. However, the Carbopol/ZnO gel showed a positive result against *C. acne*. G-Carbopol possesses a larger zone of inhibition of 15 ± 1 mm, followed by 14 ± 1 mm of C-Carbopol. The HPMC/ZnO NPs showed a similar 12 ± 1 mm zone of inhibition by both nanoparticles incorporated in the nanogel. From the results obtained, the incorporation of ZnO in the topical gel increases the surface area compared to the pure ZnO NPs, allowing a higher diffusion and interaction with the bacteria [68]. A possible factor that leads to a low zone of inhibition by pure ZnO NPs is the use of Columbia agar w/5% sheep blood. MH agar is commonly used in diffusion assays to determine the zone of inhibition, as it is classified as a ‘loose’ agar that aids in the rate of diffusion effectively [70]. The Columbia agar w/5% sheep blood is a highly nutritious medium for cultivating and isolating difficult-to-grow microorganisms and is less porous than the MH agar, leading to a lower diffusion rate [71]. Studies on anti-acne efficacy suggested the possible cultivation of CBA and MH agar under aerobic conditions; however, no growth was observed after a cultivation period of 2 weeks with consistent checking [40,72]. G-ZnO NPs exhibit a distinctive zone of inhibition against *S. aureus* of 19 ± 1 mm. Overall, the biogenic ZnO NPs showed a higher antibacterial activity than the chemogenically synthesized ZnO NPs. The nanogel-incorporated biogenically synthesized ZnO NPs simultaneously reflect a high effectiveness against acne-vulgaris-triggering bacteria. The one-way ANOVA analysis showed a similar significance (*p* < 0.001) against both bacteria using different samples of ZnO NPs.

## 4. Conclusions

*Dendrobium anosmum* contains a wide range of secondary metabolites, including carotenoids, coumarin, flavonoids, phenols, saponin, steroids, tannins, and terpenoids, identified through phytochemical screening on a leaf aqueous extract and, therefore, the determination of the total phenolic and flavonoid contents was studied. *Dendrobium anosmum* exhibited the ability to produce zinc oxide nanoparticles with the desired shape, size, and structure compared to the chemogenic synthesis technique. The physiochemical characteristics of the biogenically synthesized nanoparticles using *Dendrobium anosmum* extract were determined, which produced a smaller size, high purity, and a higher yield of the end product. The topical nanogel formulation based on Carbopol-940 and HPMC polymers showed great compatibility when combined with the chemically and biogenically synthesized ZnO NPs. Both ZnO NPs exhibited a strong antibacterial action against Gram-positive *C. acne* and *S. aureus*, with the green-synthesized ZnO NPs being superior to the chemogenically synthesized ones. The polymer and G-ZnO hybrid nanogels exhibited an increased potency against both *C. acne* and *S. aureus* due to the increased surface area when in contact with the bacteria, which led to the conclusion that the anti-acne efficacy was improved.

## Figures and Tables

**Table 1:** Phytochemical constituents of *Dendrobium anosmum* leaf crude extracts.

Phytochemical/Secondary Metabolites	Observation	*Dendrobium anosmum* Leaf Aqueous Extract
Alkaloid	No orange-red precipitate formation	−
Carotenoid	Dark blue colour at interface	+
Coumarin	Yellow colour solution	+
Flavonoids	Yellow precipitate formation	+
Phenols	Formation of bluish black colour solution	+
Phlobatannin	No red precipitate formation	−
Saponin	Formation of a persistent foam	+
Steroids	Reddish brown interface with fluorescent green with yellow	+
Tannins	White precipitate formation	+
Terpenoids	Reddish brown colour formation	+

(+) Positive detection of phytochemical compounds. (−) Negative detection of phytochemical compounds.

**Table 2:** The yield of nanoparticles obtained using chemogenic and biogenic synthesis techniques.

Nanoparticles Synthesized Technique	Weight (g)	Weight of Salt Precursor Used (g)	Yield (%)
Chemogenic	1.92 ± 0.03	12.00	15.99 ± 0.25
Biogenic	1.40 ± 0.01	2.00	69.75 ± 0.50

**Table 3:** Unit cells and crystalline size of the chemogenically and biogenically synthesized ZnO NPs.

Samples	Unit Cells		Average Crystalline Size (nm)
	A (Å)	c (Å)	
Chemogenic	3.2489	5.2049	30.40
Biogenic	3.2494	5.2058	29.15

**Table 4:** Functional groups of the transmittance bands of commercial and chemogenically and biogenically synthesized ZnO NPs.

Commercial Peaks (c)	Chemical Peak (cm^−1^)	Green Peaks (cm^−1^)	Functional Groups
3854	-	-	O-H stretching
3435	3435	3412	O-H stretching
2360	-	-	O=C=O stretching
2343	-	-	O=C=O stretching
-	2095	2066	N=N=N stretching
1636	1636	1637	C=O stretching
1383	1384	1384	N=O bending
-	-	1082	C-C stretching
-	830	871	C-H stretching
534,502	499	532,489	Zn-O stretching

**Table 5:** Physiochemical, pH, viscosity and spreadability of the nanogels.

Characteristic	Carbopol 940	HPMC
Color	Transparent	Semi-transparent
Appearance	Transparent	Cream color
Consistency	Thick and smooth	Smooth
pH	5.27 ± 0.01	5.38 ± 0.04
Viscosity (centipoises)	45,386 ± 8.29	20,000 ± 2.16
Spreadability (g·cm/s)	6.56 ± 0.24	85.23 ± 0.19

**Table 6:** Zone of inhibition exhibited by the ZnO NP and nanogel sample.

Control	Tetracycline Concentration (µg/mL)	Zone of Inhibition (mm)	
		*C. acne*	*S. aureus*
Positive	30	26 ± 1	33 ± 1
Negative	0	-	-
**Sample**	**Concentration of ZnO NPs (mg/mL)**	**Zone of inhibition (mm)**	
		***C. acne***	***S. aureus***
C-ZnO NPs	2	11 ± 1	-
	4	12 ± 1	-
	8	11 ± 1	-
	16	-	13 ± 1
G-ZnO NPs	2	12 ± 1	-
	4	12 ± 1	-
	8	10 ± 1	-
	16	10 ± 1	19 ± 1
C-Carbopol	16	14 ± 1	12 ± 1
G-Carbopol	16	15 ± 1	15 ± 1
C-HPMC	16	12 ± 1	14 ± 1
G-HPMC	16	12 ± 1	15 ± 1

## Data Availability

The data presented in this study are available upon request from the corresponding author.
